# The prevalence of depressive disorders among young people in europe: A systematic review and meta-analysis

**DOI:** 10.1192/j.eurpsy.2021.1842

**Published:** 2021-08-13

**Authors:** R. Sacco, N. Camilleri, K. Umla-Runge

**Affiliations:** 1 Psychiatry, Cardiff University, Teesside University, Malta Mental Health Services, Attard, Malta; 2 Child And Young People’s Services, Malta Mental Health Services, Pieta, Malta; 3 Psychiatry, Cardiff University, Cardiff, United Kingdom

**Keywords:** Child, Depression, prevalence, Europe

## Abstract

**Introduction:**

This systematic review estimates the pooled prevalence (PP) of depressive disorders (DD) among 5-to-18-year-old YP living in Europe, based on prevalence rates established in the last five years (LFY).

**Objectives:**

Trends of prevalence rates across countries, gender and level of education were analysed. The random effects pooled prevalence rate (REPPR) for DD was calculated.

**Methods:**

A search strategy was conducted on three databases. Studies were also identified from reference lists and grey literature. Eligible studies were evaluated for reliability, validity, bias, and the REPPR for DD was calculated.

**Results:**

The European REPPR for DD is calculated at 2.0% (95%CI: 1.0%-4.0%). (Figure 1). The REPPR for each depressive disorder is shown in Figure 1. The prevalence among secondary school children is 4.2 times higher than that among primary school children.

**Figure:**
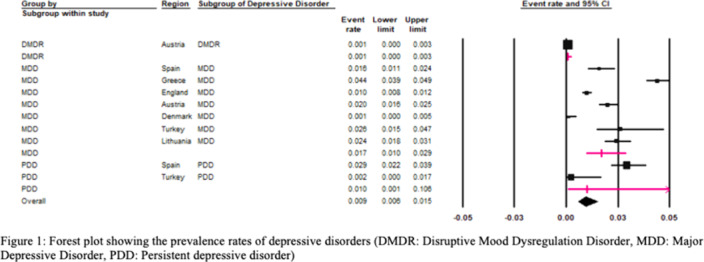

**Conclusions:**

Routine screening and early intervention strategies for eating disorders may improve the outcome of young people with these problems.

**Disclosure:**

No significant relationships.

